# Feline calicivirus and other respiratory pathogens in cats with Feline calicivirus-related symptoms and in clinically healthy cats in Switzerland

**DOI:** 10.1186/s12917-015-0595-2

**Published:** 2015-11-13

**Authors:** Alice Berger, Barbara Willi, Marina L. Meli, Felicitas S. Boretti, Sonja Hartnack, Anou Dreyfus, Hans Lutz, Regina Hofmann-Lehmann

**Affiliations:** Clinical Laboratory, Vetsuisse Faculty, University of Zurich, Zurich, Switzerland; Clinic for Small Animal Internal Medicine, Vetsuisse Faculty, University of Zurich, Zurich, Switzerland; Center for Clinical Studies, Vetsuisse Faculty, University of Zurich, Zurich, Switzerland; Section of Epidemiology, Vetsuisse Faculty, University of Zurich, Zurich, Switzerland; Present address: Clinical Laboratory, Vetsuisse Faculty, University of Zurich, Winterthurerstr. 260, 8057 Zurich, Switzerland

**Keywords:** Feline calicivirus, Feline herpesvirus-1, *Mycoplasma felis*, *Chlamydophila felis*, Risk factors, Vaccination, Upper respiratory tract disease, Gingivostomatitis, Oral ulcerations, Multivariable analysis

## Abstract

**Background:**

Cats with feline calicivirus (FCV)-related symptoms are commonly presented to veterinary practitioners. Various clinical manifestations have been attributed to FCV, i.e. upper respiratory tract disease (URTD), oral ulcerations, gingivostomatitis, limping syndrome and virulent systemic disease. Additionally, healthy cats can shed FCV. The aims of this study were 1) to investigate the frequency of FCV in cats with FCV-related symptoms and in healthy cats in Switzerland, 2) to assess risk and protective factors for infection, such as signalment, housing conditions, vaccination, and co-infection with URTD-associated pathogens, and 3) to address the association between clinical symptoms and FCV infection.

**Results:**

Oropharyngeal, nasal and conjunctival swabs were collected in 24 veterinary practices from 200 FCV-suspect and 100 healthy cats originating from 19 cantons of Switzerland. The samples were tested for FCV using virus isolation and reverse-transcription real-time quantitative polymerase chain reaction (qPCR) and for feline herpesvirus-1 (FHV-1), *Mycoplasma felis*, *Chlamydophila felis*, *Bordetella bronchiseptica* using real-time qPCR. Within the two populations (FCV-suspect/healthy), the observed PCR prevalences were: FCV 45 %/8 %, FHV-1 20 %/9 %, *C. felis* 8 %/1 %, *B. bronchiseptica* 4 %/2 %, *M. felis* 47 %/31 % and any co-infections thereof 40 %/14 %. Based on multivariable regression models amongst FCV-suspect cats (odds ratio [95 % confidence interval]), co-infection with *M. felis* (1.75 [0.97; 3.14]), group housing (2.11 [1.02; 4.34]) and intact reproductive status (1.80 [0.99; 3.28]) were found to be risk factors for FCV infection. In healthy cats, intact reproductive status (22.2 [1.85; 266.7]) and group housing (46.4 [5.70; 377.7]) were found to be associated with FCV infection. Based on an univariable approach, FCV-suspect cats were found to be significantly less often FCV-positive when vaccinated (0.48 [0.24; 0.94]). Oral ulcerations, salivation, gingivitis and stomatitis, but not classical signs of URTD were significantly associated with FCV infection (all *p* < 0.001).

**Conclusions:**

FCV was detected in less than half of the cats that were judged FCV-suspect by veterinary practitioners. For a clinical diagnosis, FCV-related symptoms should be revisited. FCV infection was present in some healthy cats, underlining the importance of asymptomatic carriers in FCV epidemiology. To reduce FCV-related problems in multi-cat environments, reduction of group size in addition to the generally recommended vaccination are advocated.

**Electronic supplementary material:**

The online version of this article (doi:10.1186/s12917-015-0595-2) contains supplementary material, which is available to authorized users.

## Background

Feline calicivirus (FCV) is a RNA virus that occurs worldwide in domestic cats and exotic felids [1, 2]. FCV infections are commonly associated with oral ulcerations and salivation [3, 4]. Other clinical syndromes that have been attributed to FCV infection include chronic stomatitis [3, 5] and a limping syndrome [6–8]. Some years ago, highly virulent systemic FCV infections associated with fatal disease have been reported in several countries, initially in North America [9, 10] and subsequently in Europe [11–13]. Outbreaks of severe FCV infections associated with edema and skin ulcerations have also been described in cats in Switzerland (Willi et al., submitted for publication).

FCV has also been assigned to the upper respiratory tract disease (URTD) complex (‘cat flu’) [14–17]. In addition to FCV, at least four other pathogens have been shown to be associated with this syndrome, including feline herpesvirus type 1 (FHV-1), *Mycoplasma felis*, *Chlamydophila felis* and *Bordetella bronchiseptica* [14, 16, 18–24]. Cats with URTD are commonly presented to veterinary practitioners and show symptoms such as lethargy, pyrexia, anorexia, sneezing, nasal discharge, ocular discharge, conjunctivitis and keratitis [14, 19].

Conventional, nested and real-time reverse-transcriptase quantitative polymerase chain reaction (RT-qPCR) assays have been developed to amplify FCV-specific RNA from clinical specimens [25–29]. The high genetic variability of FCV can hamper the diagnostic sensitivity of FCV-specific molecular assays [2]. Virus isolation has been advocated as an alternative diagnostic tool, but this method is not routinely available [2]. Virus isolation is less sensitive to genomic variation, but the method might fail due to virus inactivation during transport, thus resulting in reduced diagnostic sensitivity [2].

FCV vaccines have been shown to be efficacious in protecting cats from the development of severe disease; however, they do not induce sterilizing immunity [30, 31]. Moreover, most commercially available vaccines have been based on only a few FCV strains (i.e. FCV F9 and 255) for many decades. Therefore, the protective potential of FCV vaccines against different circulating FCV isolates has been controversially discussed in recent years [32–35]. Efforts are made for the identification of new vaccine strains with broader cross-reactivity, the development of bi- or polyvalent vaccines and the inclusion of local FCV isolates [35–38]. Along these lines, a vaccine containing two novel FCV strains has become commercially available in Switzerland (FCV G1 and 431) [35, 36].

Despite the introduction of FCV vaccines several decades ago, cats with FCV-related symptoms are still commonly presented in veterinary practices. A presumptive clinical diagnosis relies on the presence of FCV-related symptoms [2]. However, clinical signs induced by FCV and other URTD-associated pathogens can overlap and co-infections are common [4, 14–17]. Molecular assays to detect URTD-associated pathogens in clinical specimens might aid the diagnosis. Because asymptomatic carriers have been reported, results of molecular assays must be interpreted together with clinical presentation.

The aims of this study were to investigate the frequency of FCV in cats suspected FCV infected by veterinary practitioners and in clinically healthy cats in Switzerland, and to address potential risk and protective factors for infection in both groups of cats, such as signalment, housing conditions, vaccination, and co-infection with URTD-associated pathogens. Furthermore, the association between a number of different clinical symptoms and FCV infection was assessed.

## Methods

### Cats

**Fig. 1:**
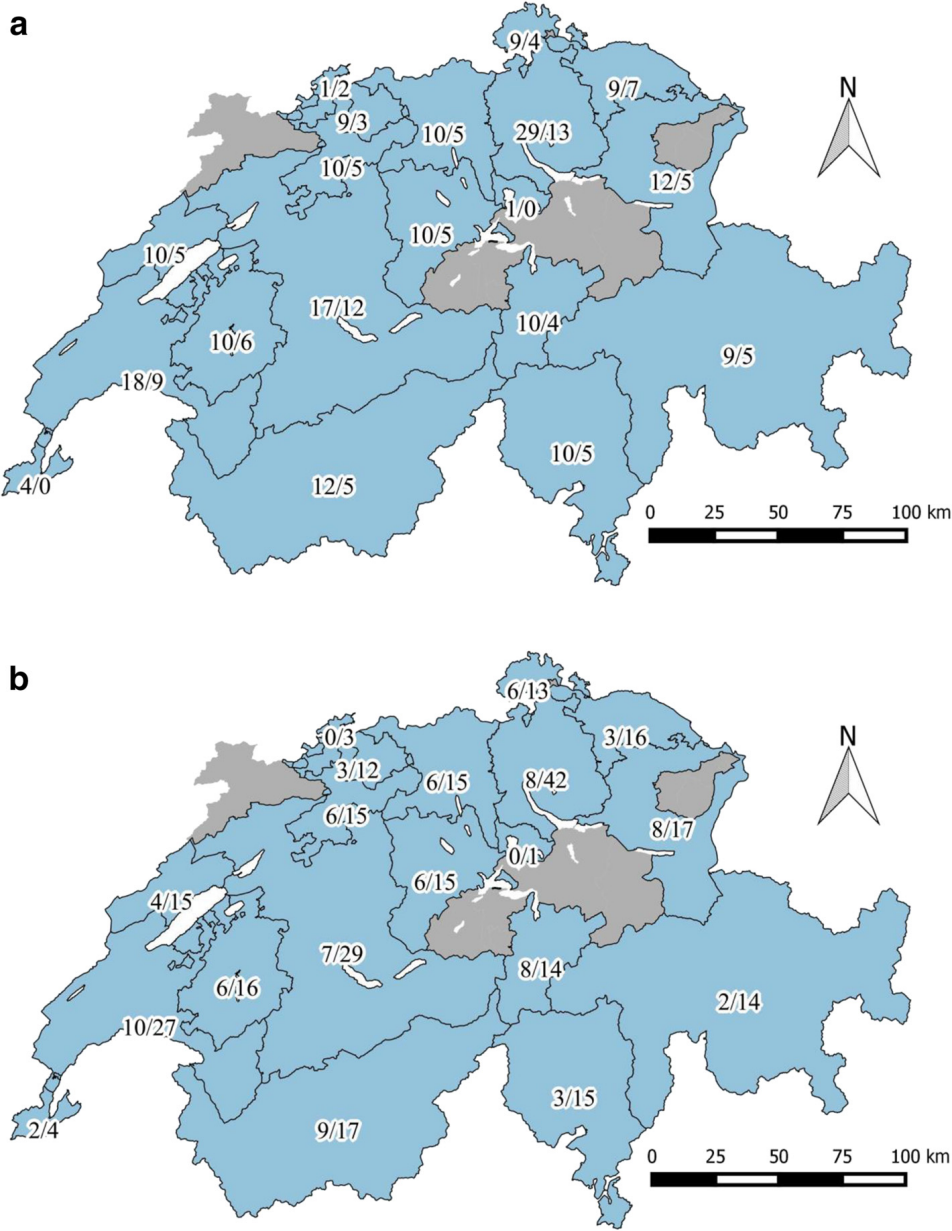
Map of Switzerland depicting the origin of the 300 cats enrolled in the study. The numbers listed give the number of cats per canton: **a** FCV-suspect/healthy cats; **b** FCV-positive/all cats. Basel-Landschaft and Basel-Stadt are listed as a single canton. Maps were produced using QGIS [51]

A total of 200 clinically diseased cats with symptoms compatible with FCV infection (FCV-suspect cats) and 100 clinically healthy cats were enrolled. The study was part of a large FCV project with the goal to obtain a collection of FCV isolates from symptomatic and asymptomatic cats in Switzerland for genetic and serological studies. Both populations were sampled by veterinary surgeons in Switzerland between September 2012 and April 2013. The goal was to include samples originating from 20 randomly selected veterinary practices in 17 different geographic regions (cantons) of Switzerland (ten FCV-suspect and five healthy cats per practice). Only one cat per owner (e.g., same household, cattery) was included. The veterinarians obtained informed consent from the cat owners. All samples were taken as part of a diagnostic workup or for routine testing of FeLV/FIV in healthy cats and all results were provided to the veterinarians and the cat owners; no ethical approval was necessary for this study in compliance with Swiss regulations [39]. Veterinary practices that were not able to collect a sufficient number of samples within the given time frame (eight months) were supported by a second or third veterinary practice within the same canton. This resulted in the participation of 24 veterinary practices in 17 cantons and the enrollment of 300 cats originating from 19 out of 26 cantons in Switzerland (Fig. 1).

The participating veterinarians were provided with a list of the following FCV-related clinical signs: sneezing, nasal and/or ocular discharge, conjunctivitis, caudal stomatitis, chronic stomatitis and/or gingivitis, lingual and/or oral ulceration, pneumonia, shifting lameness, or a combination of fever, cutaneous edema and dermal ulceration of the face and/or limbs. The clinical signs were selected based on the guidelines on feline calicivirus infection by the European Advisory Board on Cat Diseases (ABCD) and a recent publication [2, 40]. The written instructions contained illustrations on how to recognize FCV-associated clinical signs, as described above, and how to collect and ship the samples. Inclusion criteria for clinically healthy cats were the absence of any clinical signs based on anamnesis and clinical examination of the participating veterinarian. Cats that had been vaccinated against FCV, FHV-1 or *C. felis* within 21 days prior to the sample collection or that were younger than eight weeks were excluded.

To record demographic data for each enrolled animal, the veterinarians filled out a questionnaire during the cat’s visit at the clinic. To avoid interviewer bias, the participating veterinarians received written instructions on how to fill in the questionnaire. A similar questionnaire has been used in earlier studies [41, 42]. The questionnaire was comprised of different sections on geographic data (address of the cat owner), demographic data (age, sex, reproductive status, breed of the cat), husbandry data (type of husbandry, such as private home, farm, cat breeder, feral cat and cattery, number of cats per household, outdoor access, contact to dogs with kennel cough), data on vaccine history according to the clinical record and vaccination card (vaccination status for FCV/FHV-1/feline parvovirus, date of primary immunization, booster immunizations, vaccines used, vaccination status for feline leukemia virus (FeLV), date of the last FeLV vaccination), retrovirus status (FeLV and/or feline immunodeficiency virus (FIV) test results and date of testing), data on medical history (antiviral, antibiotic and immunosuppressive treatment at the time of sample collection and up to two months before collection) and results of the clinical examination.

### Sample collection and analysis

An oropharyngeal cytobrush, as well as a nasal and a conjunctival cotton swab were collected from each cat. Sample collection material was provided by the investigators. Conjunctival swabs were collected prior to any diagnostic treatment, such as fluorescein application. Each cytobrush and swab was placed in a sterile Safe-Lock Eppendorf tube containing 300 μL of sterile viral transport medium. The medium consisted of 200 mL bi-distilled sterile water, 4 mL HEPES-Buffer 1 M (Sigma-Aldrich Chemie GmbH, Steinheim, Germany), 25 mL Dulbecco’s MEM 10x (Biochrom, Berlin, Germany), 25 mL heat inactivated fetal calf serum (Charge DO2303P, Origin South America, Bio Concept, Allschwil, Switzerland), 3 mL 100 × Antibiotic-Antimycotic (Gibco, Life Technologies, Lucerne, Switzerland) and 4 mL sodium hydrogen bicarbonate 7.5 % (Merck, Darmstadt, Germany) at a pH of 7 that was adjusted using 1 M sodium hydroxide (Merck). Samples were stored at 4 °C prior to shipping to the laboratory by priority mail. All samples were processed within 96 h after collection.

### Sample processing and total nucleic acid (TNA) extraction

Upon arrival, the cytobrushes and swabs were incubated at 40 °C for 10 min. The cytobrushes and swabs were then turned upside down and centrifuged for 1 min at 6440 × g. The samples from each patient (oropharyngeal cytobrush and nasal and conjunctival swabs) were pooled and subsequently divided as follows: two aliquots of 200 μL were used for TNA extractions and 400 μL were used for virus isolation. TNA extraction was performed using 200 μL of sample or cell culture supernatant with the MagNa Pure LC (Roche Diagnostics AG, Rotkreuz, Switzerland) using the MagNa Pure LC Total Nucleic Acid Isolation Kit (Roche Diagnostics) following the manufacturer’s instructions. In each batch of extraction, a negative control that consisted of 200 μL of PBS was used to monitor for cross-contamination. TNA was stored at -20 °C until qPCR analysis.

### Virus isolation

For virus isolation, 400 μL of each sample were filtered (Filtropur S 0.45 μm syringe filter, Sarstedt, Nümbrecht, Germany), incubated on 80 % confluent Crandell-Rees feline kidney cells in 24-well plates (TPP Tissue Culture Testplate 24, TPP Techno Plastic Products AG, Trasadingen, Switzerland) and cultured using RPMI 1640 Medium (Sigma-Aldrich Chemie GmbH) supplemented with 10 % heat inactivated fetal calf serum (Invitrogen, Basel, Switzerland), 2 mM L-Glutamin (Gibco, Life Technologies) and 1x Antibiotic-Antimycotic (Gibco, Life Technologies). For each sample culture, a negative medium-only control was run in parallel. The samples were incubated on cells for two hours before 300 μL of complete medium were added. The cells were fed daily and evaluated for the presence of a cytopathic effect. As soon as a cytopathic effect became visible or after a maximum of seven days, the supernatants were collected for TNA extraction and for storage at −80 °C until further use. If the TNA extraction could not be performed on the day of collection, 300 μL Lysis Buffer (MagNa Pure LC Total Nucleic Acid Isolation Kit, Roche Diagnostics) were added to the 200 μL of supernatant and the mixture was stored at −20 °C as recommended by the manufacturer. TNA was extracted from the cell culture supernatants after a maximum of 72 h of storage.

### Real-time qPCR and RT-qPCR assays

All real-time qPCR assays in the current study were run with 5 μL TNA in an end volume of 25 μL. Positive and negative controls were run with each RT-qPCR and qPCR assay. All oligonucleotides were synthetized by Microsynth AG (Balgach, Switzerland), and all qPCR assays were run on an ABI 7500Fast Real-Time PCR system (Applied Biosystems, Rotkreuz, Switzerland). For quantitative analyses, CT values were used.

For FCV, two previously described FCV real-time TaqMan RT-qPCR assays were used [25, 26]. The assays were optimized prior to the start of the experiment. The FCV RT-qPCR S1 [25] reaction contained 1 x One step RT-qPCR MasterMix Low ROX (Eurogentec, Seraing, Belgium), 300 nM forward primer, 900 nM reverse primer, 250 nM probe, 5 μL nuclease-free water (Gibco, Life Technologies) and 0.125 μL Euroscript (Eurogentec). The temperature profile was 30 min at 48 °C, followed by 10 min at 95 °C and 45 cycles of 15 s at 95 °C, followed by 1 min at 60 °C. The FCV RT-qPCR S2 [26] reaction contained 0.5 μL Superscript III/RT Platinum Taq Mix (Invitrogen, Life technologies), 300 nM forward primer, 900 nM reverse primer, 250 nM probe, 0.05 μL 2.5x Rox dye, 0.625 μL 40 U/μL rRNasin® RNase Inhibitor (Promega, Dübendorf, Switzerland) or RNasin® Plus RNase Inhibitor (Promega) and 3.95 μL nuclease-free water (Gibco, Life Technologies). The temperature profile was 30 min at 50 °C, followed by 2 min at 95 °C and 45 cycles of 20 s at 95 °C, followed by 45 s at 60 °C. Each sample (TNA from swab/cytobrush and TNA from cell culture supernatant) was analyzed with both RT-qPCR assays (S1 and S2). All cats that tested positive in at least one of the four RT-qPCR runs were categorized as FCV-positive (FCV RT-qPCR S1 or RT-qPCR S2 from swabs/cytobrush or from virus isolation).

TNA from swab/cytobrush from each cat was also tested for FHV-1 [43], *C. felis* [44], *B. bronchiseptica* [15] and *M. felis* [45] by qPCR using previously described methods. Moreover, the samples were also tested for FeLV [46] and FIV viral RNA [47] by RT-qPCR because both infections can lead to increased susceptibility to other infections and can be associated with inflammatory oral disease. Three samples from FCV-suspect cats could not be analyzed for *B. bronchiseptica*, *M. felis*, FeLV and FIV because of a lack of material.

### Statistical analysis

Statistical analysis was performed utilizing the freely available software program R version 3.2.0 [48]. Separately for the FCV-suspect and clinically healthy cats, descriptive statistics with frequencies for male and female cats, medians with 95 % confidence interval (CI) for age and proportions of the dichotomous variables with 95 % CI based on the binomial distribution are presented. Regression models were applied to assess if the predictors (age in years, sex: male or female, reproductive status: intact or neutered, pedigree: yes or no, outdoor access, living in groups of at least four cats, being vaccinated at all or having received a primary immunization, having received immunosuppressive, antimicrobial and/or antiviral treatment or being co-infected with *M. felis*, FHV-1, *C. felis* and/or *B. bronchiseptica*) were significantly associated with the outcome FCV infection. Univariable regressions, with the outcome FCV were performed and odds ratios with 95 % Wald confidence intervals are shown. Due to the presence of missing values which reduced the number of complete cases (meaning cats with information for all predictors) for FCV-suspect animals to 168 and for clinically healthy animals to 88, missing values were imputed with the package missForest [49], separately for the FCV-suspect and clinically healthy cats. Imputation was done iteratively based on a random forest approach. Utilizing the imputed data sets, multivariable logistic regressions were performed with the package MASS [50]. Models were built following two different approaches. First, all variables with a *p*-value < 2 in univariable regression were considered in different models, including interaction terms. Model selection was based on AIC (Akaike’s information criterion) with lower values of at least 2 considered indicative of a better model fit. In case of between model differences smaller than 2, the most parsimonious model was chosen. Second, a stepwise regression approach in both directions was applied including all variables, irrespective of their *p*-values in the univariate approach and interaction terms. Diagnostic plots were assessed for the robustness of the final models. Results are presented as odds ratios and 95 % Wald confidence intervals. FCV loads in swab/cytobrush samples were compared between two groups using the Mann–Whitney *U*-test (p_MWU_) and the Graph-Pad Prism Version 3.0 (GraphPad Software, San Diego, CA, USA). Maps were produced using QGIS Geographic Information System (v. 2.6.1) [51]. Canton boundaries were provided by the Swiss Federal Office of Topography.

## Results

Summary statistics for all variables used in the statistical analysis for FCV-suspect and healthy cats are presented in Table 1. This includes the PCR results for URTD-associated pathogens. FCV infection was detected in 45 % (95 % CI 38–52 %) of the FCV-suspect cats and in 8 % (95 % CI 4–15 %) of the healthy cats. Among the 200 FCV-suspect and 100 healthy cats, 40 % (95 % CI 33 – 47 %) and 14 % (95 % CI 8 – 22 %) respectively, were co-infected with any of the tested URTD-associated pathogens. In summary, among the co-infected FCV-suspect cats, 77 % were positive for two, 21 % for three and 3 % for four pathogens. Among the co-infected healthy cats, 93 % were positive for two and 7 % for three pathogens. For details see Additional file 1. In 26 % (95 % CI 20–33 %) of the FCV-suspect cats, none of the tested URTD-associated pathogens were detected. Five cats (four FCV-suspect and one healthy cat, 1.7 %, 95 % CI 0.4–5 %) tested positive either for FeLV or for FIV. Because of the low prevalence, FIV and FeLV infections were not considered in the statistical analysis. Approximately two thirds of the cats lived in a group with other cats (Table 1); details of the numbers of cats per group and the frequency of FCV infection are shown in Additional file 2. The majority of the FCV-suspect (76 %, 95 % CI 69–82 %) and healthy cats (80 %, 95 % CI 70–87 %) were vaccinated; of these 92 % of the FCV-suspect (95 % CI 86–96 %) and the healthy cats (95 % CI 83–97 %) had received a primary immunization defined as a minimum of two vaccinations two to six weeks apart with the same vaccine strain (Table 1). In both groups of cats, the vaccine strain most frequently used for primary immunization was FCV F9 (FCV-suspect cats: 81 %, 95 % CI 73–88 %; healthy cats: 86 %, 95 % CI 75–93 %) followed by FCV G1/431 (FCV-suspect cats: 10 %, 95 % CI 5–18 %; healthy cats: 11 %, 95 % CI 5–22 %). Because of the small number of cats vaccinated with a vaccine strain other than F9, vaccine strain was not considered in the statistical analysis.

**Table 1:** Characteristics of the 200 FCV-suspect and the 100 healthy cats. The cats originated from 19 different cantons in Switzerland

Parameter	FCV-suspect cats (*n* = 200)			Healthy cats (*n* = 100)		
	Median	Lower 95 % CI^a^	Upper 95 % CI^a^	Median	Lower 95 % CI^a^	Upper 95 % CI^a^
Age (years)	4	0.20	16	0.75	0.3	14.8
	Proportion	Lower 95 % CI^a^	Upper 95 % CI^a^	Proportion	Lower 95 % CI^a^	Upper 95 % CI^a^
Sex (male)	0.60	0.52	0.66	0.61	0.51	0.71
Intact reproductive status	0.37	0.30	0.44	0.40	0.30	0.50
Pedigree	0.32	0.25	0.39	0.22	0.14	0.32
Breed^bc^						
Maine Coon	0.09	0.05	0.14	0.02	0.01	0.07
British Shorthair	0.05	0.02	0.09	0.02	0.01	0.07
Norwegian Forest Cat	0.05	0.02	0.09	0.02	0.01	0.07
Persian	0.04	0.02	0.08	0.04	0.01	0.1
Siamese	0.03	0.01	0.06	0.02	0.01	0.07
Multi-cat household	0.72	0.65	0.78	0.63	0.53	0.72
Group housing with ≥ 4 cats	0.22	0.16	0.29	0.16	0.09	0.25
Outdoor access	0.57	0.50	0.64	0.58	0.48	0.68
Contact with dog with kennel cough^c^	0.02	0	0.05	0.01	0	0.06
Vaccinated	0.76	0.69	0.82	0.80	0.70	0.87
Primary immunization^d^	0.69	0.61	0.75	0.72	0.61	0.81
Immunosuppressive therapy	0.15	0.10	0.20	0.01	0.00	0.05
Antibiotic therapy	0.42	0.35	0.49	0.02	0.00	0.07
Antiviral therapy	0.05	0.02	0.08	0.00	0.00	0.04
FCV positive^e^	0.45	0.38	0.52	0.08	0.04	0.15
*M. felis* positive^e^	0.48	0.41	0.55	0.31	0.22	0.41
FHV-1 positive^e^	0.20	0.15	0.26	0.09	0.04	0.16
*C. felis* positive^e^	0.08	0.05	0.13	0.01	0.00	0.05
*B. bronchiseptica* positive^e^	0.04	0.02	0.08	0.02	0.00	0.07

^a^CI = Confidence interval. ^b^Only breeds with >5 cats in the study are listed. ^c^Due to low numbers, these variables were not considered in the statistical analysis. ^d^Primary immunization was defined as two subsequent vaccinations within 2 to 6 weeks with the same vaccine strain. ^e^Positive by real-time qPCR/RT-qPCR

Based on univariable regression models, in FCV-suspect cats intact reproductive status, living in a group of at least four cats, co-infection with *M. felis* and both vaccination and primary immunization were found to be significantly associated with FCV infection (Table 2). The former three predictors were found to be risk factors by approximately doubling the chance of a FCV infection (if being intact versus neutered, if living in a group of at least four cats and if being co-infected with *M. felis)*. Being vaccinated at all or having received a primary immunization were found to be protective factors by reducing the chance of a FCV infection by approximately 50 %. In contrast, amongst the clinically healthy cats, the vaccination predictors were not significantly associated with FCV infection (Table 2). Similarly to the FCV-suspect cats, intact reproductive status and living in a group of at least four cats were found to be risk factors, albeit with higher odds ratios and considerably larger confidence intervals than in the FCV-suspect cats. Details on missing values, univariable regressions with complete case and imputed data sets are presented in Additional file 3. Whereas *p*-values obtained with the original data and the imputed data are similar, *p*-values from complete case analyses differ slightly.

**Table 2:** Results of the univariable approach for the 200 FCV-suspect and the 100 healthy cats

Parameter	FCV-suspect cats (*n* = 200)				Healthy cats (*n* = 100)			
	*p*-value	Odds ratio	Lower 95 % CI^a^	Upper 95 % CI^a^	*p*-value	Odds ratio	Lower 95 % CI^a^	Upper 95 % CI^a^
Age	0.12				0.70			
Sex	0.38				0.51			
Intact reproductive status	**0.02**	**2.02**	**1.13**	**3.62**	**0.02**	**12.52**	**1.48**	**106.20**
Pedigree	0.87				0.28			
Multi-cat household	0.09	1.76	0.92	3.37	0.17			
Group housing with ≥ 4 cats	**0.008**	**2.63**	**1.29**	**5.37**	**<0.001**	**26.33**	**4.61**	**150.42**
Outdoor access	0.19				0.23			
Vaccinated	**0.03**	**0.48**	**0.24**	**0.94**	0.57			
Primary Immunization^b^	**0.03**	**0.49**	**0.26**	**0.92**	0.83			
Immunosuppressive therapy	0.40				1.00			
Antibiotic therapy	0.26				0.99			
Antivirale therapy	0.51				NA^c^			
*M. felis* positive^d^	**0.02**	**2.03**	**1.15**	**3.59**	0.06	4.23	0.94	18.99
FHV-1 positive^d^	0.78				0.99			
*C. felis* positive^d^	0.27				0.99			
*B. bronchiseptica* positive^d^	0.74				0.99			

Parameters significantly associated with FCV infection are shown in bold. Odds ratios and confidence intervals are given for *p* < 0.1

^a^CI = Confidence interval . ^b^Primary immunization was defined as two subsequent vaccinations within 2 to 6 weeks with the same vaccine strain. ^c^Not applicable. ^d^Positive by real-time qPCR/RT-qPCR

Based on multivariable regression models, in FCV-suspect animals only intact reproductive status, living in a group of at least four cats and co-infection with *M. felis* remained in the final model with all odds ratios being reduced by at least 10 % compared to the univariable results (Table 3). Based on the stepwise regression with all predictors, outdoor access was additionally found to be a risk factor for FCV infection (data not shown). None of the variables describing vaccination were found to be significant in the multivariable models. Regarding the clinically healthy cats, intact reproductive status and living in a group of at least four cats remained in the final model with higher odds ratios and larger confidence intervals than in the FCV-suspect cats. Stepwise regression was not possible for this group of cats due to lack of convergence.

**Table 3:** Results from multivariable regressions for the 200 FCV-suspect and the 100 healthy cats

Parameter^a^	FCV-suspect cats (*n* = 200)			Healthy cats (*n* = 100)		
	Odds ratio	Lower 95 % CI^b^	Upper 95 % CI^b^	Odds ratio	Lower 95 % CI^b^	Upper 95 % CI^b^
Intact reproductive status	1.80	0.99	3.28	22.22	1.85	266.73
Group housing with ≥ 4 cats	2.11	1.02	4.34	46.39	5.70	377.72
*M. felis* positive^c^	1.75	0.97	3.14			

Only parameters significantly associated with FCV infection are shown

^a^Based on the stepwise regression with all predictors, additionally outdoor access was found to be a risk factor for FCV infection. ^b^CI = confidence interval. ^c^Positive by real-time qPCR

**Fig. 2:**
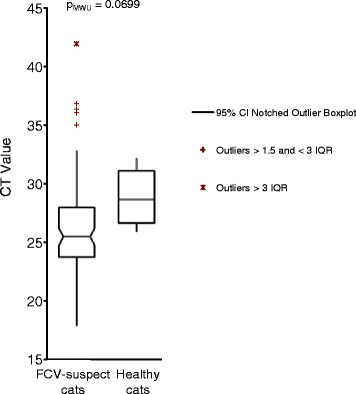
Comparison of FCV loads in the swab/cytobrush samples from FCV-suspect cats and the healthy cats. Loads are given as CT values from the real-time RT-qPCR S1 assay and are depicted as boxplots. A low CT value corresponds to a high load. Of note, the measurements are semi-quantitative because of the collection procedure (cytobrushes and swabs)

The clinical signs in the 200 cats chosen by the veterinary practitioners because of FCV-related symptoms are shown in Table 4. In approximately half of the FCV-suspect cats, gingivitis or classical signs of URTD, such as nasal discharge, ocular discharge, conjunctivitis and sneezing were present. Approximately one third of the cats exhibited stomatitis or caudal stomatitis, about 10 % demonstrated oral or lingual ulceration and only a few were found with swollen joints, lameness, skin ulcerations or cutaneous edema. Gingivitis, stomatitis, caudal stomatitis, salivation and oral and lingual ulcerations were significantly associated with FCV infection (Table 4). In contrast, nasal and ocular discharge and sneezing were significantly less common in the FCV PCR-positive than in the FCV PCR-negative cats. When the FCV load in the sample material was compared between FCV-positive diseased and FCV-positive healthy cats, the FCV loads were higher in the diseased cats, although statistical significance was not reached (FCV S1 RT-qPCR; p_MWU_ = 0.07; Fig. 2).

**Table 4:** Association of clinical signs with FCV infection in the 200 FCV-suspect cats

Clinical signs	Number of FCV-suspect cats	Number of FCV-positive cats	Number of FCV-negative cats	Odds ratios	Lower 95 % CI^a^	Upper 95 % CI^a^
	(*n* = 200)	(*n* = 89)	(*n* = 111)			
Gingivitis	103	62	41	**3.89**	**2.08**	**7.43**
Stomatitis	59	43	16	**5.50**	**2.71**	**11.64**
Caudal stomatitis	61	40	21	**3.48**	**1.78**	**6.95**
Salivation	38	30	8	**6.48**	**2.69**	**17.47**
Oral ulceration	23	18	5	**5.33**	**1.80**	**19.22**
Lingual ulceration	20	15	5	**4.27**	**1.40**	**15.67**
Nasal discharge	112	38	74	**0.37**	**0.20**	**0.69**
Ocular discharge	102	34	68	**0.39**	**0.21**	**0.72**
Sneezing	84	27	57	**0.41**	**0.22**	**0.77**
Keratitis	10	3	7	0.52	0.08	2.36
Conjunctivitis	93	39	54	0.82	0.45	1.50
Anorexia	49	23	26	1.14	0.56	2.29
Apathy	47	21	26	1.01	0.49	2.05
Elevated body temperature	29	14	15	1.19	0.50	2.84
Lameness	7	5	2	3.23	0.51	34.67
Skin ulcerations	6	4	2	2.55	0.36	28.85
Joint swelling	2	2	0	6.37	0.30	134.5
Cutaneous edema	2	1	1	1.25	0.08	20.28

Significant associations are shown in bold

^a^CI = confidence interval

## Discussion

The present study investigated FCV infection in 200 diseased cats judged suspicious for FCV infection by private veterinarians and in 100 clinically healthy cats, all sampled in different regions of Switzerland. FCV infection was detected in 45 % (95 % CI 38 – 52 %) of the cats with FCV-related symptoms. This relatively low percentage of PCR-positive symptomatic cats is in agreement with the prevalence of FCV infection in cats in UK with respiratory disease (33 %, 95 % CI 27–39 %) [19] and in cats in Germany with chronic gingivostomatitis (54 %, 95 % CI 39–68 %) [5]. In the present study, cats were judged as FCV-suspect by the veterinary practitioners based on written instructions that listed FCV-related symptoms as described in the literature [2, 40]. Interestingly, cats suffering from gingivitis and URTD-related symptoms were most commonly selected by the veterinarians. However, only gingivitis, stomatitis, caudal stomatitis, salivation and oral and lingual ulcerations were significantly associated with FCV infection but not the classical signs of URTD, such as sneezing, nasal and ocular discharge. In contrast, sneezing, nasal and ocular discharge were significantly less common in the FCV-positive cats when compared to the FCV-negative cats. This finding contrasts earlier studies where FCV infection was significantly associated with feline respiratory tract disease [14, 15, 19, 52]. However, in accordance with our results, Bannasch and co-workers found that among the five pathogens associated with URTD examined (the same as in the present study), the most important contributors to URTD were FHV-1, *Mycoplasma* species and *C. felis* [16]. Acute FCV is known to induce vesicular disease, which typically manifests as oral and lingual ulcerations [53]. Ocular and nasal discharge is only rarely reported after experimental FCV infection [6, 36, 54]. Chronic FCV infection has been strongly associated with the presence of gingivostomatitis [5], although the exact pathogenesis of this disease is still unknown. Our data suggest that for a clinical diagnosis of FCV infection, the FCV-related symptoms should be reconsidered and more stringently defined and the diagnosis confirmed by molecular testing.

It needs to be noted that the relatively low detection rate of FCV in the FCV-suspect cats could also be attributed to a limited diagnostic sensitivity of the applied diagnostic assays. In the current study, the criterion of being FCV-positive was based on the presence of at least one positive out of four real-time RT-qPCR runs (two different PCR assays performed directly from the sample material and after an enrichment step in cell culture). This approach was chosen to maximize diagnostic sensitivity. However, the remarkably high genetic variability of FCV [55, 56] increases the likelihood of potential mismatches in the primer or probe binding sites, thus leading to the possibility of failed real-time PCR amplification.

Interestingly, 26 % of the FCV-suspect cats tested negative for all URTD-associated pathogens. Cats with a non-infectious etiology for URTD (i.e., neoplasia, foreign bodies or primary immune-mediated rhinitis) could account for some of these cases. Cats with non-infectious URTD are clinically indistinguishable from cats with URTD caused by infectious agents. Additionally, the detection of the URTD-associated pathogens could have failed due to sample degradation during the transport from the veterinary practices to the laboratory [2]. To avoid or minimize this effect, the samples in the present study were analyzed within 96 h of collection. Finally, the diagnostic sensitivity of the PCR assays could have been hampered by genetic alterations of the pathogens within the target sequence of the applied PCR assays. However, in contrast to RNA viruses such as FCV, a high genetic variability is not necessarily expected for FHV-1, *C. felis*, *M. felis* and *B. bronchiseptica*.

Approximately 8–9 % of the healthy cats in the present study tested PCR-positive for FHV-1 and FCV. This proportion is lower for FCV than reported in earlier studies in the UK (22 %, 95 % CI 17–29 %) [19] and in Germany (14 %, 95 % CI 6 – 27 %) [5]. As previously reported [52], it is important to remember that some cats, although clinically healthy, are shedders of infectious pathogens related to URTD.

In the present study, risk factor analysis was performed with univariable and multivariable regression models. The latter were performed with imputed data sets due to missing data. The selected multivariable regression approach offers, in contrast to univariable approaches, the possibility to adjust for confounding. Inevitably however, it remains possible that not all relevant confounders were recorded. Missing values leading subsequently to different sample sizes of models with different variables, preclude the possibility to compare the model fit: thus data sets with imputed variables for the missing values were generated. Although resulting *p*-values obtained with the original and the imputed data sets were similar in univariable analysis, any imputation may potentially include bias. Two model-building approaches were performed for the FCV-suspect cats (the stepwise approach was not possible for the clinically healthy cats) to assure robustness of the final models.

Although found to be significant protective factors in univariable models, statistical association for vaccination status and primary immunization vanished in multivariable analysis. One reason might be that the predictors “vaccination status” and “primary immunization” with the categories “yes” and “no” (or “unknown” in 18 and 8 cases for the FCV-suspect and healthy cats respectively) comprised a number of different vaccines, vaccinations schemes and time elapsed since vaccination, thus, potentially being too heterogeneous to actually show a statistical association. In addition, FCV vaccines do not induce sterilizing immunity, although they have been shown to reduce the severity of clinical signs [30, 31]. Therefore, vaccine protection cannot be solely judged by the PCR status of a cat. Furthermore, a cross-sectional study is rather limited in assessing causal relationships, especially since in a cross-sectional approach only prevalence can be estimated whereas incidence would be more relevant.

An intact reproductive status, living in a group with at least four cats and co-infection with *M. felis* were found to be risk factors for FCV infection. All of these three factors approximately doubled the chance of finding a FCV infection. A number of interaction terms, including interaction between groups of at least four cats and *M. felis* infection or between vaccination and intact reproductive status, were tested for potential association but none was found to be statistically significant.

An association of FCV with intact reproductive status has been reported in an earlier study [52]. Although no clear explanation can be given for this association, possible reasons postulated earlier include hormonal effects, which might alter the replication and the persistence of the virus, and more social or aggressive interactions between the intact animals [52].

The association of FCV infection with housing in large groups of cats was reported in previous studies [34, 57]. Cats kept in large groups are exposed to a higher infectious pressure compared with cats living in smaller groups. The high plasticity of the FCV genome and positive selection by the immune system support genomic mutations and lead to a progressive evolution of FCV variants in cat groups with cyclic reinfection of previously immune cats [58, 59]. In addition, strict quarantine measures and excellent hygiene can be more challenging to guarantee in large groups of cats, thereby favoring FCV infections [15]. Breeders and other cat owners should be educated about the benefits of housing cats in small groups. A reduced group size does not only reduce the risk of infection related to URTD in cats but also the risk of other feline infections and the development of feline infectious peritonitis [60, 61].

No clear explanation can be given for the association of FCV infection with *M. felis* co-infection. Possible reasons could be a similar transmission route, pathogenesis or similar risk factors for infection. FCV and FHV-1 infection have often been reported to occur concurrently, and cats co-infected with FCV and FHV-1 had a high risk of developing URTD [62]. However, no association between FCV infection and co-infections with the other tested pathogens FHV-1, *C. felis* and *B. bronchiseptica* were found in the present study.

The different housing categories including private home, farm, breeder, feral cat and cattery were recorded in this study, but since the vast majority of cats was kept in private homes, it was not possible to disentangle the effect of different housing categories. The variable breed was dichotomized into pedigree and non-pedigree cats for statistical analysis because the number of cats per breed was small. The absence of a statistical association does therefore not preclude any potential association between specific breeds and FCV infection. For example out of 17 FCV-suspect Maine Coons, 13 were found to be FCV-infected and suffer from gingivostomatitis. It is known that purebreds, such as the Maine Coons, are predisposed to develop early or severe periodontal disease [63], but no association with FCV infection has been described thus far. It should be noted however that 12 of the FCV-positive Main Coons were housed in groups (data not shown).

In the present study, 76 % and 80 % of the FCV-suspect and healthy cats, respectively, were vaccinated against FCV and FHV-1 (i.e. had received some vaccinations against FCV and FHV-1 at any time point). This vaccination rate seems higher than the rate estimated for Switzerland in 2009 according to the number of sold vaccine doses (30 to 40 % of all Swiss cats vaccinated) [64]. Thus, according to our numbers, the proportion of vaccinated cats would be sufficiently high to provide some protection for the population (herd immunity) within the sampled population as opposed to protection of the vaccinated host only [65]. However, the present study is based on preselected populations of cats and some of the cats had not received a proper primary immunization or were lacking the necessary booster vaccinations, leaving some doubts about the actual level of protection.

## Conclusion

The present study assessed FCV infection in cats judged FCV-suspect by veterinary practitioners and in clinically healthy cats in Switzerland. FCV infection was detected in less than half of the FCV-suspect cats. Within this group of cats, infection was significantly associated with gingivitis, stomatitis, caudal stomatitis, oral and lingual ulcerations, but not with classical signs of URTD, such as sneezing, nasal and ocular discharge. These results suggest that the FCV-related symptoms should be critically reassessed and probably more stringently defined. FCV infection was also detected in some healthy cats; this observation underlines the importance of asymptomatic shedders in FCV epidemiology. Housing in large groups of cats was among the risk factors for FCV infection. Thus, if problems with FCV are encountered in multi-cat environments, reduction of group size in addition to the generally recommended vaccination and quarantine measures for incoming and sick cats are advocated.

## Additional files

Additional file 1:
**Number of cats PCR-positive for URTD-associated pathogens and co-infections thereof.** The 200 FCV-suspect and 100 healthy cats originated from 19 different cantons in Switzerland. (PDF 7 kb) Additional file 2:
**Frequency of FCV-positive and FCV-negative cats according to number of cats housed per group.** a) 200 FCV-suspect cats; b) 100 healthy cats. FCV-pos./neg. = FCV-positive/negative in FCV real-time RT-PCR. Number of cats = 1: single cats. The numbers in the bars represent the absolute numbers of cats in the respective category. (PDF 82 kb) Additional file 3:
**Details on missing values, univariable regressions with complete case and imputed data sets.** (PDF 45 kb)

## Abbreviations

*B. bronchiseptica*: *Bordetella bronchiseptica*
*C. felis*: *Chlamydophila felis*
CI: confidence interval
FCV: Feline calicivirus
FeLV: Feline leukemia virus
FHV-1: Feline herpesvirus-1
FIV: Feline immunodeficiency virus
*M. felis*: *Mycoplasma felis*
qPCR: quantitative polymerase chain reaction
RT-qPCR: reverse-transcriptase qPCR
TNA: total nucleic acid
URTD: upper respiratory tract disease
