# Supplementary material for: Feline calicivirus and other respiratory pathogens in cats with Feline calicivirus-related symptoms and in clinically healthy cats in Switzerland
Source: BMC Vet Res. 2015 Nov 13;11:282. doi: 10.1186/s12917-015-0595-2 (PMC4644299; doi:10.1186/s12917-015-0595-2)
Supplement: Additional file 1: — Number of cats PCR-positive for URTD-associated pathogens and co-infections thereof. The 200 FCV-suspect and 100 healthy cats originated from 19 different cantons in Switzerland. (PDF 7 kb) [file 12917_2015_595_MOESM1_ESM.pdf]

Additional file 1: Number of cats PCR-positive for URTD-associated pathogens and co-infections thereof. *The 200 FCV-suspect and 100 healthy cats originated from 19 different cantons in Switzerland.*

(RT-) qPCR-positive for					Number of cats	
**FCV**	***M. felis***	**FHV-1**	***C. felis***	***B. bronchi-septica***	**FCV-suspect cats (n = 200)**	**Healthy cats (n = 100)**
+	+	+	+	+	0	0
+	+	+	+	-	1	0
+	+	+	-	+	0	0
+	+	+	-	-	11	0
+	+	-	+	+	1	0
+	+	-	+	-	1	1
+	+	-	-	+	2	0
+	+	-	-	-	34	4
+	-	+	+	+	0	0
+	-	+	+	-	0	0
+	-	+	-	+	1	0
+	-	+	-	-	4	0
+	-	-	+	+	0	0
+	-	-	+	-	2	0
+	-	-	-	+	0	1
+	-	-	-	-	32*	2
-	+	+	+	+	0	0
-	+	+	+	-	0	0
-	+	+	-	+	1	0
-	+	+	-	-	10	8
-	+	-	+	+	0	0
-	+	-	+	-	6	0
-	+	-	-	+	0	0
-	+	-	-	-	27	18
-	-	+	+	+	0	0
-	-	+	+	-	1	0
-	-	+	-	+	2	0
-	-	+	-	-	9**	1
-	-	-	+	+	1	0
-	-	-	+	-	3	0
-	-	-	-	+	0	1
-	-	-	-	-	51	64

* Contains two FCV-suspect cats, which could not be tested for *B. bronchiseptica* and *M. felis* because of a lack of material. ** Contains one FCV-suspect cat, which could not be tested for *B. bronchiseptica* and *M. felis* because of a lack of material.
