# Supplementary material for: Feline calicivirus and other respiratory pathogens in cats with Feline calicivirus-related symptoms and in clinically healthy cats in Switzerland
Source: BMC Vet Res. 2015 Nov 13;11:282. doi: 10.1186/s12917-015-0595-2 (PMC4644299; doi:10.1186/s12917-015-0595-2)

Additional file 2: Frequency of FCV-positive and FCV-negative cats according to number of cats housed per group*. a) 200 FCV-suspect cats; b) 100 healthy cats. FCV-pos./neg. = FCV-positive/negative in FCV real-time RT-PCR. Number of cats = 1: single cats. The numbers in the bars represent the absolute numbers of cats in the respective category.*

a)

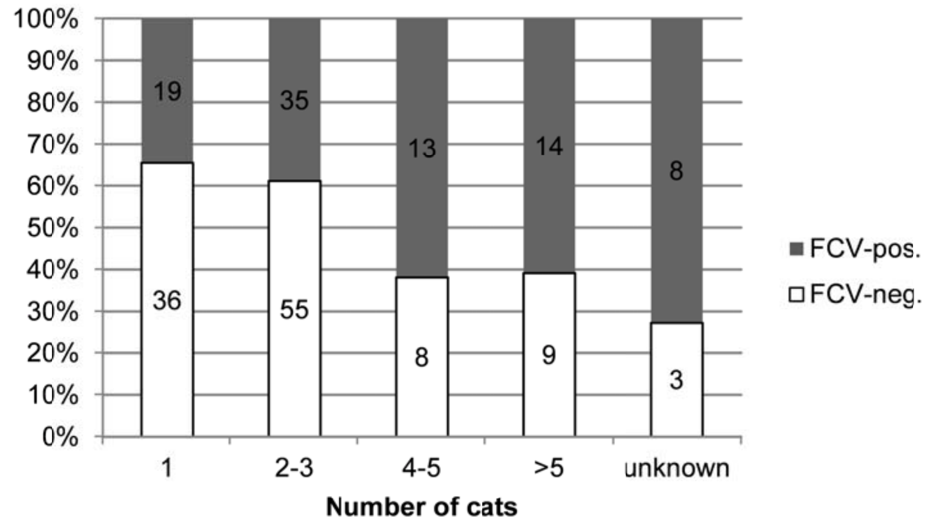

b)

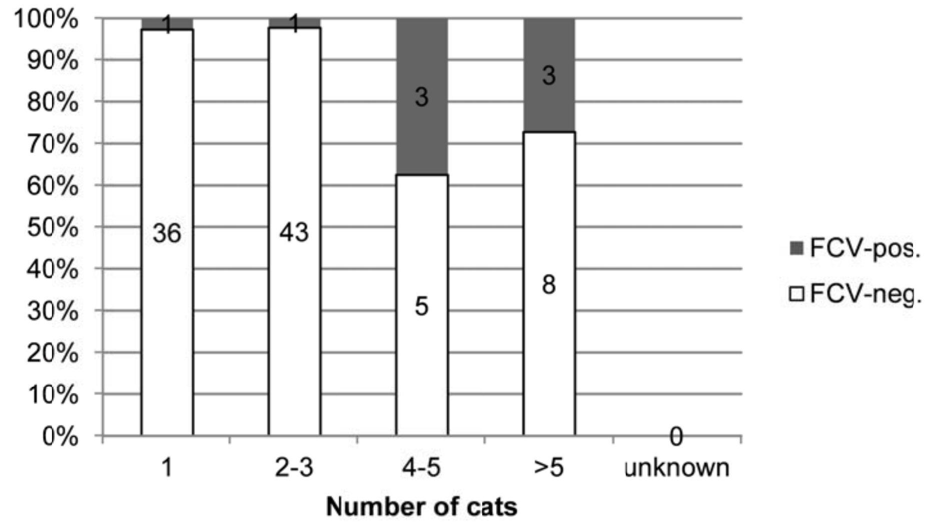

Supplement: Additional file 2: — Frequency of FCV-positive and FCV-negative cats according to number of cats housed per group. a) 200 FCV-suspect cats; b) 100 healthy cats. FCV-pos./neg. = FCV-positive/negative in FCV real-time RT-PCR. Number of cats = 1: single cats. The numbers in the bars represent the absolute numbers of cats in the respective category. (PDF 82 kb) [file 12917_2015_595_MOESM2_ESM.pdf]
