# Supplementary material for: Feline calicivirus and other respiratory pathogens in cats with Feline calicivirus-related symptoms and in clinically healthy cats in Switzerland
Source: BMC Vet Res. 2015 Nov 13;11:282. doi: 10.1186/s12917-015-0595-2 (PMC4644299; doi:10.1186/s12917-015-0595-2)
Supplement: Additional file 3: — Details on missing values, univariable regressions with complete case and imputed data sets. (PDF 45 kb) [file 12917_2015_595_MOESM3_ESM.pdf]

Additional file 3: Details on missing values, univariable regressions with complete case and imputed data sets

Parameter	FCV-suspect (n = 200)		FCV-suspect, complete (n = 168)	FCV-suspect, imputed (n = 200)	Healthy (n = 100)		Healthy, complete (n = 88)	Healthy, imputed (n = 100)
	**p-value**	**missing**	**p-value**	**p-value**	**p-value**	**missing**	**p-value**	**p-value**
Age	0.12	-	0.03	0.12	0.70	1	0.76	0.71
Sex	0.38	-	0.42	0.38	0.51	-	0.45	0.51
Intact reproductive status	0.02	-	0.03	0.02	0.02	-	0.01	0.02
Pedigree	0.87	-	0.98	0.87	0.28	-	0.35	0.28
Multi-cat household	0.09	6	0.11	0.08	0.17	-	0.17	0.17
Group housing with ≥ 4 cats	0.008	6	0.009	0.009	< 0.001	4	< 0.001	< 0.001
Outdoor access	0.19	1	0.17	0.17	0.23	-	0.387	0.23
Vaccinated	0.03	8	0.03	0.029	0.57	2	0.67	0.55
Primary Immunization ‡	0.03	18	0.04	0.039	0.83	8	1.00	0.86
Immunosuppressive therapy	0.40	-	0.90	0.399	0.995	-	1.00	1.00
Antibiotic therapy	0.26	-	0.32	0.256	0.99	-	0.99	0.99
Antivirale Therapy	0.51	1	0.52	NA†	NA†		NA†	NA†
FHV-1 positive*	0.77	-	0.82	0.776	0.99	-	0.99	0.99
C. felis positive*	0.27	-	0.43	0.27	0.99	-	0.99	0.99
B. bronchiseptica positive*	0.74	3	0.58		0.99	-	0.99	0.99
M. felis positive*	0.02	3	0.13	0.019	0.06	-	0.07	0.06

‡ Primary immunization defined as two subsequent vaccinations within 2 to 6 weeks with the same vaccine strain. † Not applicable. * Positive by PCR / RT-PCR.
